# Revisiting the “Paradox of Stereotaxic Surgery”: Insights Into Basal Ganglia-Thalamic Interactions

**DOI:** 10.3389/fnsys.2021.725876

**Published:** 2021-08-27

**Authors:** Jennifer L. Magnusson, Daniel K. Leventhal

**Affiliations:** ^1^Department of Neurology, University of Michigan, Ann Arbor, MI, United States; ^2^Department of Biomedical Engineering, University of Michigan, Ann Arbor, MI, United States; ^3^Parkinson Disease Foundation Research Center of Excellence, University of Michigan, Ann Arbor, MI, United States; ^4^Department of Neurology, VA Ann Arbor Health System, Ann Arbor, MI, United States

**Keywords:** deep brain stimulation (DBS), Parkinson disease, rate model, local field potential (LFP), thalamus

## Abstract

Basal ganglia dysfunction is implicated in movement disorders including Parkinson Disease, dystonia, and choreiform disorders. Contradicting standard “rate models” of basal ganglia-thalamic interactions, internal pallidotomy improves both hypo- and hyper-kinetic movement disorders. This “paradox of stereotaxic surgery” was recognized shortly after rate models were developed, and is underscored by the outcomes of deep brain stimulation (DBS) for movement disorders. Despite strong evidence that DBS activates local axons, the clinical effects of lesions and DBS are nearly identical. These observations argue against standard models in which GABAergic basal ganglia output gates thalamic activity, and raise the question of how lesions and stimulation can have similar effects. These paradoxes may be resolved by considering thalamocortical loops as primary drivers of motor output. Rather than suppressing or releasing cortex via motor thalamus, the basal ganglia may modulate the timing of thalamic perturbations to cortical activity. Motor cortex exhibits rotational dynamics during movement, allowing the same thalamocortical perturbation to affect motor output differently depending on its timing with respect to the rotational cycle. We review classic and recent studies of basal ganglia, thalamic, and cortical physiology to propose a revised model of basal ganglia-thalamocortical function with implications for basic physiology and neuromodulation.

## Basal Ganglia Anatomy and the Standard “Rate Model”

**FIGURE 1:**
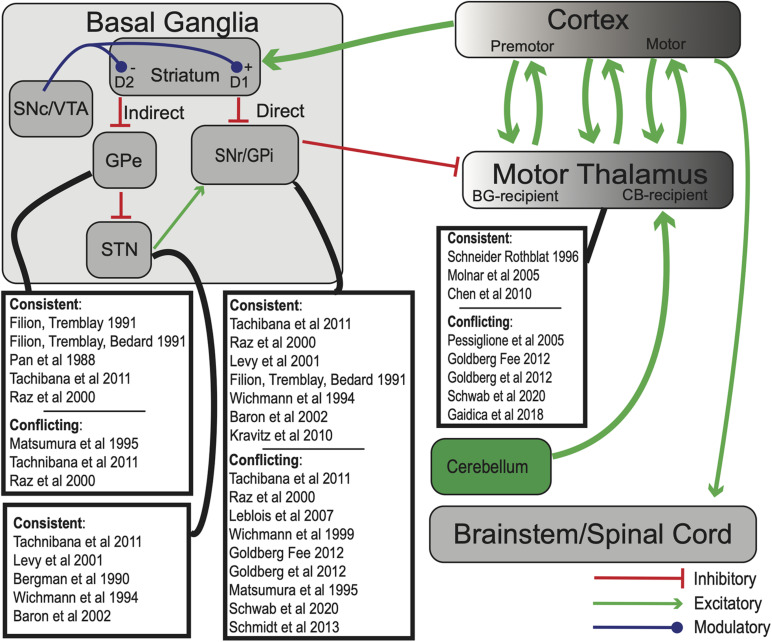
Simplified model of BG-thalamocortical interactions. In standard “rate models” of BG-thalamocortical function, competing direct and indirect pathways determine SNr/GPi firing rates, which in turn suppress or release BG-recipient motor thalamic regions to drive movement. Green arrows—excitatory (glutamatergic) projections; red—inhibitory (GABAergic) projections, blue—dopaminergic. Experiments whose results are consistent or conflict with rate model predictions are highlighted for each BG nucleus. Note that some experiments may have results that both support and contradict the rate model, even in the same nucleus. Abbreviations are defined in the text.

The basal ganglia (BG) are heavily interconnected subcortical nuclei spanning the midbrain, diencephalon, and telencephalon (Figure 1). The striatum consists of GABAergic medium spiny projection neurons (MSNs) (Wilson and Groves, 1980) as well as several types of interneurons (Tepper et al., 2004, 2010), and is the primary receptive nucleus of the BG. It receives input from most areas of the neocortex, specific thalamic nuclei, and dopaminergic neurons of the substantia nigra pars compacta (SNc) and ventral tegmental area (VTA) (Parent et al., 1983; Kreitzer and Malenka, 2008). Serotonergic afferents from the dorsal raphe (Ternaux et al., 1977), noradrenergic afferents from the locus coeruleus (Mason and Fibiger, 1979), and more recently, cholinergic afferents from the pedunculopontine nucleus (PPN) (Dautan et al., 2020) have also been identified. The primary output nuclei of the BG are the GABAergic globus pallidus pars interna (GPi) and substantia nigra pars reticulata (SNr), which project to the BG-recipient zone of “motor” thalamus (BG-Mthal). BG-Mthal connects primarily with premotor cortex, forming reciprocal loops that interact with primary motor cortex and the cerebellar-recipient thalamus (CB-Mthal) (McFarland and Haber, 2002; Haber and Calzavara, 2009; Tanaka et al., 2018). In the rest of this manuscript, we use the terms BG- and CB-Mthal to avoid confusion due to differing nomenclature across species, and the term “Mthal” when there is ambiguity regarding which subregion was studied. In addition to the canonical motor BG-thalamocortical circuit, the mediodorsal and centromedian-parafascicular nuclear complex (CM-pf) of the thalamus receive input from motor cortices and the BG. The mediodorsal nucleus forms reciprocal connections with prefrontal cortex, while CM-pf projects to striatum, pallidum, the subthalamic nucleus (STN) and cortex (Lanciego et al., 2009; Ilyas et al., 2019; McElvain et al., 2021).

Standard “rate” models of BG function describe two routes of information transmission from the striatum to BG output nuclei: the “direct” and “indirect” pathways. According to the model, direct pathway dopamine D1-receptor expressing GABAergic MSNs disinhibit the thalamus by suppressing the GABAergic GPi and SNr. The indirect pathway originates from dopamine D2-expressing MSNs that project to the globus pallidus pars externa (GPe; in rodents typically referred to simply as GP). D2-MSNs are believed to decrease GPe output, which increases STN activity, increases GPi activity, and suppresses BG-Mthal (Albin et al., 1989; DeLong, 1990; Gerfen et al., 1990). Because D1 receptor activation increases MSN excitability and D2 receptor activation decreases MSN excitability (Gerfen and Surmeier, 2011), the direct and indirect pathways are inversely modulated by dopamine. Ultimately, firing rates of thalamocortical neurons in BG-Mthal regulate motor cortical activity, which in turn regulates movement “vigor” (i.e., movement frequency, amplitude, or velocity). Direct pathway activation therefore promotes movement, while indirect pathway activation suppresses movement. At the level of BG-thalamic interactions, the model predicts that increased BG output should decrease movement vigor, and decreased BG output should have the opposite effect.

## Evidence Supporting “Rate Models”

Hypotheses regarding the role of the BG in motor function have been strongly influenced by observations in human disease. This is especially true of Parkinson Disease (PD) because its neurochemistry and pathology are relatively well understood. PD is characterized by slowness of movement (bradykinesia), increased muscular tone, tremor, and postural instability. Rate models predict that bradykinesia results from underactive direct and overactive indirect pathways due to decreased D1 and D2 receptor activation, respectively. This should result in decreased GPe, BG-Mthal, and motor cortical activity; and hyperactive STN and BG output nuclei (GPi/SNr). These predictions are largely supported by single-unit recordings in non-human primates (NHPs) rendered parkinsonian by MPTP (Filion and Tremblay, 1991; Schneider and Rothblat, 1996; Raz et al., 2000; Pasquereau and Turner, 2011; Tachibana et al., 2011) and 6-OHDA-treated rodents (Pan and Walters, 1988). PD patients also have elevated GPi and reduced BG-Mthal firing rates compared to other neurologic disorders (Molnar et al., 2005; Starr et al., 2008; Chen et al., 2010). Furthermore, dopamine agonists both improve symptoms and alter BG firing rates in ways consistent with rate models (Filion et al., 1991; Levy et al., 2001; Parker et al., 2018). Finally, manipulations that reduce STN discharge like permanent lesions (Bergman et al., 1990) or transient inactivation (Wichmann et al., 1994; Baron et al., 2002; Tachibana et al., 2011) improve parkinsonism.

Observations in hyperkinetic disorders also influenced the development of rate models (Albin et al., 1989). D2-receptor bearing indirect pathway MSNs degenerate early in Huntington Disease (Reiner et al., 1988; Albin et al., 1992), which is characterized by excessive abnormal involuntary movements. Reduced indirect pathway activity should reduce BG output, releasing thalamic suppression. STN lesions in healthy subjects induce contralateral hemiballismus (large, “flinging” involuntary limb movements) (Carpenter et al., 1950; Crossman, 1987), which similarly is attributed to depressed downstream activity in BG output nuclei. Because these observations were used to create the model, however, they do not represent independent validation of its predictions.

Optogenetics made it possible to directly test rate model predictions in mice. By selectively expressing an excitatory opsin (channelrhodopsin, ChR2) in D1− or D2-MSNs, blue light could activate one population independently of the other. Selective activation of D1-MSNs decreased SNr activity, while D2-MSN activation increased SNr activity (Kravitz et al., 2010). Behaviorally, D2-MSN activation suppressed movement, and D1-MSN activation rescued locomotion in 6-OHDA-treated mice.

Collectively, these data support the notion that, at least under certain conditions, the rate model predicts behavioral and physiologic outcomes of neuronal manipulations.

## Evidence Contradicting “Rate Models”

Not all single-unit recordings are consistent with rate model predictions. For example, BG output firing rates did not change significantly in symptomatic MPTP-treated NHPs (Wichmann et al., 1999; Raz et al., 2000; Leblois et al., 2007; Tachibana et al., 2011). Furthermore, MPTP-treated NHPs did not exhibit firing rate changes in primary motor cortex (Goldberg et al., 2002) or BG-Mthal (Pessiglione et al., 2005; Kammermeier et al., 2016). BG-Mthal firing rates increased in 6-OHDA lesioned rats, again directly contradicting rate model predictions (Bosch-Bouju et al., 2014). While some single-unit recordings match rate model predictions, it is difficult to attribute clinical phenomenology to firing rate changes if they do not always match model predictions.

**FIGURE 2:**
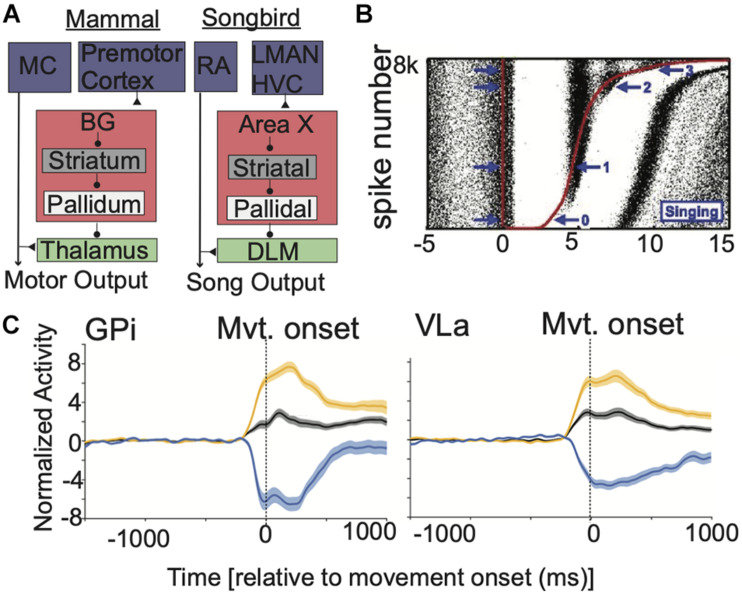
BG output alters the timing of BG-Mthal spikes but not their average rate. **(A)** Comparative anatomy between mammalian and songbird BG-thalamocortical loops. Reproduced with permission from Goldberg et al. (2012). **(B)** Pallidal spikes regulate the fine timing of thalamic (DLM) spikes during birdsong. Red dots indicate pallidal spikes, rasters indicate thalamic spikes from a connected thalamic neuron. Trials are sorted by the duration of the pallidal interspike interval. Note that thalamic spiking is briefly suppressed after each pallidal spike, aligning thalamic spikes across trials but without reducing their overall firing rate on a longer time-scale. Reproduced with permission from Goldberg et al. (2012). **(C)** In NHPs performing reaches, response patterns are similar in GPi and VLa (an Mthal subregion with pallidal afferents). Population-averaged spike-density functions are shown for all neurons (black) for subpopulations that increase (orange) or decrease (blue) firing rates near movement onset. Shaded regions indicate SEM. Reproduced with permission from Schwab et al. (2020).

Furthermore, BG output and BG-Mthal activity increase concurrently at movement onset (Goldberg and Fee, 2012; Schwab et al., 2020). By itself, this does not necessarily contradict rate models. Subsets of disinhibited BG-Mthal neurons could initiate a desired action, while BG output suppresses other BG-Mthal neurons that drive alternative movements (an “action selection” model) (Redgrave et al., 1999; Maia and Frank, 2011; Mink, 2018; Logiaco et al., 2021). This idea is difficult to test directly, but several lines of evidence suggest that this is not the case. Songbirds have a unique anatomy in which large calyceal synapses (Luo and Perkel, 1999) allow simultaneous recording of thalamic (dorsolateral division of the medial thalamus—DLM) neurons and their pallidal (Area X) input (Person and Perkel, 2007). Inhibitory pallidal inputs and thalamic neurons increase activity concurrently during zebra finch song (Goldberg and Fee, 2012). If the pallidum suppresses alternative actions while thalamus activates a specific song, highly active pallidal terminals should suppress their connected thalamic neurons while less active terminals allow their postsynaptic neurons to fire. Instead, presynaptic pallidal terminal activity suppressed connected thalamic neurons for only ∼5 ms, altering action potential timing in their postsynaptic neurons (Figure 2B). However, the average firing rate of connected thalamic neurons increased over a timescale of seconds. While this anatomy is specific to songbirds, it suggests that pallidal activity alters fine thalamic spike timing without necessarily decreasing mean BG-Mthal firing rates (at least, under physiologic conditions in healthy animals).

Recording pallidal afferents and their specific thalamic single neuron targets is extremely difficult, if not impossible in mammals *in vivo*. Nonetheless, simultaneous GPi and BG-Mthal recordings are inconsistent with rate model predictions. In NHPs performing reaches, not only were there similar peri-movement rate changes in the GPi and BG-Mthal (Figure 2), but changes in GPi firing lagged changes in BG-Mthal (Schwab et al., 2020). Furthermore, cross-correlation functions between individual pallidal and thalamic neurons were rarely significant. When significant cross-correlations were found, they were generally inconsistent with monosynaptic pallidal-thalamic inhibition (i.e., pallidal spikes were not followed by a decrease in thalamic firing probability). These data further argue against pallidal gating of thalamic activity.

The most compelling argument that rate models are incomplete is the “paradox of stereotaxic surgery,” which was identified soon after rate models were introduced (Marsden and Obeso, 1994). Consistent with rate model predictions, pallidotomy improves parkinsonism (Svennilson et al., 1960; Laitinen et al., 1992; Lozano et al., 1995; Baron et al., 1996, 2000; Bastian et al., 2003). However, pallidotomy also improves hyperkinetic disorders including dystonia (Lozano et al., 1997; Vitek and Bakay, 1997) and chorea, including levodopa-induced dyskinesias (Lozano et al., 1995; Lang et al., 1997; Jankovic et al., 1999). Rate models predict that all of these should be exacerbated by pallidal inactivation. Furthermore, pallidal-recipient thalamic lesions in NHPs do not cause permanent bradykinesia, if they cause bradykinesia at all (Canavan et al., 1989). On the other hand, GPi lesions or inactivation consistently slow movement in previously healthy subjects, again in direct opposition to rate model predictions (Horak and Anderson, 1984; Mink and Thach, 1991; Inase et al., 1996; Desmurget and Turner, 2010). Furthermore, “action selection,” as assessed by the ability to recall overlearned sequences, is preserved with pallidal inactivation (Desmurget and Turner, 2010). Interestingly, bradykinetic PD patients move faster after pallidotomy, but patients with preserved movement speed move slower (Bastian et al., 2003).

## Deep Brain Stimulation

Deep brain stimulation (DBS) treats bradykinesia, rigidity, and tremor in PD by delivering continuous, high-frequency electrical stimulation to GPi or STN (Starr et al., 1998). The clinical effects of high-frequency DBS present another paradox: they are very similar to the effects of lesions at the same locations. This was initially interpreted to mean that DBS works by suppressing neural activity, possibly by depolarization blockade (Burbaud et al., 1994; Benazzouz et al., 1995; Beurrier et al., 2001). Modeling studies indicated that local somata should be hyperpolarized by DBS (McIntyre et al., 2004), and neuronal recordings showed that somatic activity is suppressed during high frequency stimulation (Boraud et al., 1996; Dostrovsky et al., 2000; Dostrovsky and Lozano, 2002; Chiken and Nambu, 2013). However, the same models also predict that axons, whether afferent, efferent, or axons of passage, should be activated by DBS. Furthermore, single-unit recordings and neurochemical measurements downstream from stimulation sites are more consistent with activation than suppression of efferent fibers (i.e., stimulation in a glutamatergic/GABAergic nucleus mostly increases/decreases downstream neuronal activity, respectively) (Windels et al., 2000; Hashimoto et al., 2003; Vitek et al., 2004; Muralidharan et al., 2017). Finally, high frequency optogenetic STN stimulation improves parkinsonism in 6-OHDA-treated rats, which is difficult to attribute to somatic suppression (Yu et al., 2020). Collectively, these data argue that there is a second “paradox of stereotaxic surgery”—that neuronal suppression and high frequency activation have nearly identical clinical effects.

The outcomes of DBS coupled with the evidence cited above has led to widespread recognition that the rate model is incomplete, but an adequate replacement has remained elusive.

## Physiologic Changes Associated With Parkinsonism

The development of animal models of PD and the advent of DBS allowed direct comparisons of physiology between healthy and diseased states. As it became clear that the rate model is incomplete, it also became possible to identify physiologic changes in parkinsonism.

While both tremor and bradykinesia/rigidity are clearly linked to dopamine loss, they have distinct pathophysiologic mechanisms. Several lines of evidence link tremor to changes in both BG- and cerebello-thalamic circuits. DBS of the cerebellar-recipient thalamus (the ventral intermediate nucleus, VIM) improves parkinsonian tremor, but not bradykinesia or rigidity (Lyons et al., 2001; Hariz et al., 2008). However, parkinsonian tremor also responds (usually) to levodopa, as well as subthalamic and pallidal DBS (Weaver et al., 2012; Wong et al., 2020) [though it has been argued that STN DBS may improve tremor by activating passing cerebello-thalamic fibers (Abdulbaki et al., 2021)]. Single-unit spike-tremor coherence in both VIM (Magnin et al., 2000) and the STN (Amtage et al., 2008) also suggest that both BG- and cerebello-thalamic circuits participate in parkinsonian tremor. Precisely how striatal dopamine loss leads to downstream changes in both BG- and cerebellar-thalamic circuits to generate tremor remains unclear, however. Several hypotheses have been advanced, including effects of extrastriatal (especially thalamic) dopamine loss (Sanchez-Gonzalez et al., 2005; Dirkx et al., 2017), interactions between BG and cerebellar circuits at the level of thalamocortical circuits (Helmich et al., 2021), and transmission of aberrant BG signaling to the cerebellum via disynaptic subthalamo-cerebellar connections (Bostan and Strick, 2018).

In contrast to tremor, BG-thalamic interactions are more directly implicated in the pathogenesis of bradykinesia and rigidity. Three themes have emerged: enhanced neuronal oscillations, synchrony, and burst-firing, though controversy remains regarding the relative importance of each in causing parkinsonism.

Enhanced “beta” (∼13–30 Hz, though definitions vary) oscillations are consistently observed in parkinsonism, both in single unit firing and local field potential (LFP) oscillations. MPTP-treated non-human primates have enhanced oscillatory firing among single units in the STN and GPi (Bergman et al., 1994). These findings were corroborated in DBS patients, who showed dramatic decreases in subthalamic LFP beta power with levodopa treatment and motor symptom improvement (Brown et al., 2001). Beta oscillations are robustly and consistently correlated with bradykinesia/rigidity in parkinsonism (Kuhn et al., 2006; Ray et al., 2008; Neumann et al., 2016), and suppressed during effective treatment (Kuhn et al., 2006; Weinberger et al., 2006; Ray et al., 2008; Feldmann et al., 2021; Kehnemouyi et al., 2021). Furthermore, subthalamic (Eusebio et al., 2008; Chen et al., 2011) or cortical (Guerra et al., 2021) stimulation at beta frequencies slows movement.

However, beta oscillations are not a purely pathological phenomenon. Brief bursts of beta oscillations occur in healthy subjects throughout BG-thalamocortical circuits (Leventhal et al., 2012; Feingold et al., 2015; Shin et al., 2017; Gaidica et al., 2020). Behaviorally, beta bursts are associated with tonic muscle contraction (Baker et al., 1997), active movement suppression (Swann et al., 2009), and slowed reaction times (Leventhal et al., 2012) and movement speed (Gilbertson et al., 2005). Conversely, beta bursts are less likely during movement (Sanes and Donoghue, 1993; but see Leventhal et al., 2012). Amphetamine makes rats hyperactive, and also suppresses beta oscillations (Berke, 2009). Collectively, these data suggest that beta oscillations normally stabilize the current behavioral state (Engel and Fries, 2010; Shin et al., 2017). In PD, beta bursts are prolonged, increasing beta amplitude in power spectra averaged over long timescales (Tinkhauser et al., 2017, 2018; Deffains et al., 2018; Pina-Fuentes et al., 2019; Eisinger et al., 2020; Yu et al., 2021). Dopamine loss may prolong “normal” beta events, preventing transitions to new behaviors. However, it is also possible that “normal” beta events are distinct from the pathological beta of parkinsonism. Models suggest thalamocortical (Sherman et al., 2016), striatal (McCarthy et al., 2011), or subthalamo-pallidal (Mirzaei et al., 2017) origins for normal and pathologic beta. It remains unclear if a solitary beta phenomenon is caused by one mechanism, or if several mechanisms cause distinct beta phenomena.

While it is possible that beta oscillations are simply a biomarker of parkinsonism (Weinberger et al., 2009), there are plausible mechanisms through which they could influence behavior. In healthy subjects, single unit activity is phase-locked to transient beta oscillations in cortex (Murthy and Fetz, 1996; Reimer and Hatsopoulos, 2010) and throughout the basal ganglia (Leventhal et al., 2012). This phase locking is enhanced after dopamine loss (Kuhn et al., 2005; Yang et al., 2014; Deffains et al., 2016). In addition, phase-amplitude coupling between beta and high frequency (∼300 Hz) LFP oscillations in the STN (Lopez-Azcarate et al., 2010), GPi (Connolly et al., 2015b; Tsiokos et al., 2017), and cortex (de Hemptinne et al., 2013) is higher in the dopamine-depleted state and correlated with bradykinesia and rigidity (de Hemptinne et al., 2015). These high frequency LFP oscillations may reflect multi-unit activity (Meidahl et al., 2019), further suggesting that large groups of neurons across structures are increasingly entrained to beta oscillations as dopamine is lost. It is suggested that this network-wide synchrony reduces the information coding capacity of BG-thalamocortical circuits, leading to decreased behavioral flexibility (i.e., inability to initiate movement) (Brittain and Brown, 2014). However, this still does not fully explain why periodic synchronized neuronal activation at beta frequencies should alter movement speed.

## An Alternative View of BG-Thalamocortical Interactions

Corticothalamic projections are often omitted from “box and arrow” diagrams of BG-thalamocortical circuits, but are necessary and sufficient to generate movement-locked modulation of BG-Mthal activity. In songbirds, thalamic (DLM) neurons exhibit similar song-linked modulation in intact and pallidal (Area X)-lesioned finches (Goldberg and Fee, 2012). Furthermore, changes in corticothalamic activity precede song-locked DLM modulation. Muscimol injected in GPi of NHPs caused only mild changes in movement-linked BG-Mthal modulation (Inase et al., 1996). In mice, anterior lateral motor cortex (ALM, homologous to human premotor cortex) and BG-Mthal activity are mutually dependent (Guo et al., 2017). During the delay period in a forced choice task, ALM and BG-Mthal activity ramp up and predict impending choice. ALM inhibition reduces BG-Mthal activity, and vice versa. It may be more appropriate to consider the BG as modulating ongoing cortico-thalamocortical activity instead of gating actions by suppressing and releasing BG-Mthal. This is more consistent with modern views of other thalamocortical circuits. Instead of simple relays for ascending sensory signals or cortico-cortical communication, it is suggested that thalamocortical circuits regulate and maintain activity across cortical networks (Halassa and Kastner, 2017).

**FIGURE 3:**
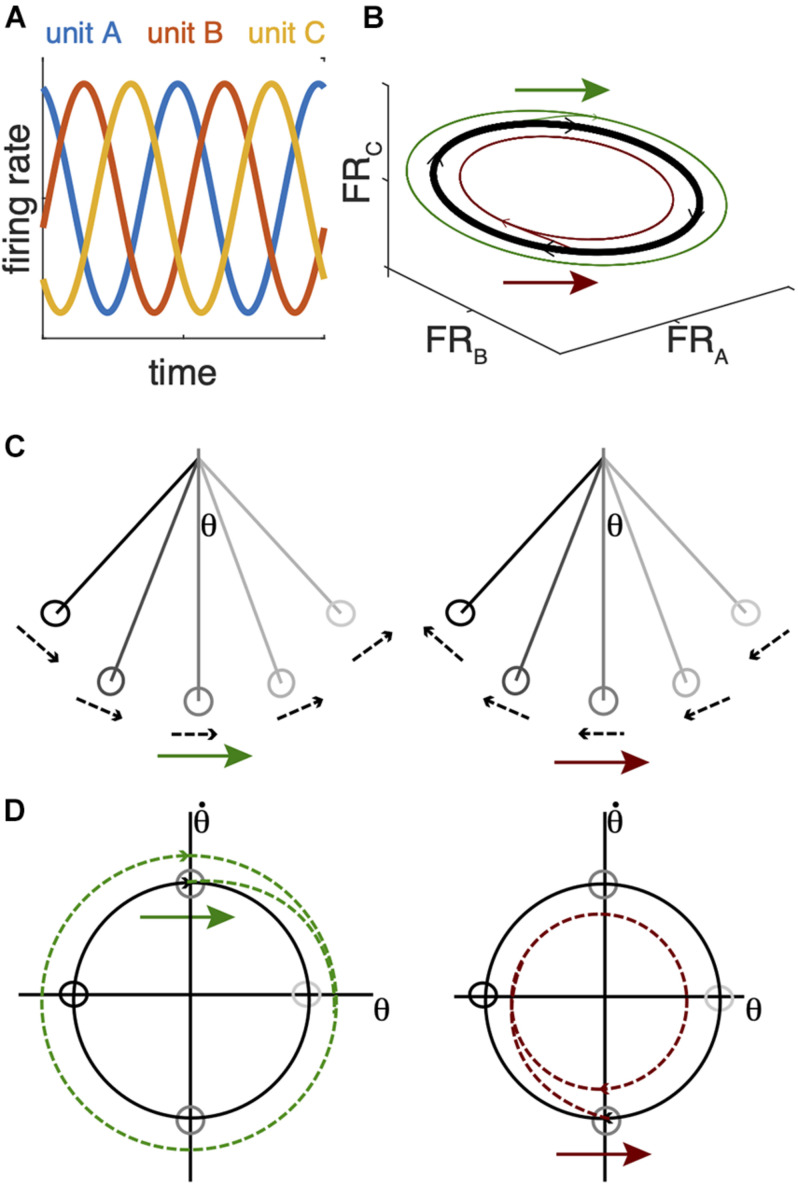
Analogy between a simple pendulum and cortical firing rate state-space dynamics. **(A)** Idealized periodic changes in firing rates for 3 single units. **(B)** The firing patterns of the units in **(A)** trace an elliptical orbit in the firing rate state space. In an actual recording, more units would be recorded, and many more would be unobserved. Population dynamics are often analyzed with a dimensionality-reduction technique to project the high dimensional firing rate state space (one dimension per neuron) into a subspace that accounts for most of the variance in the recorded signal (e.g., Churchland et al., 2012). **(C)** A simple pendulum also traces a rotating trajectory in the state space defined by its angular position (θ) and velocity **(D)**. The same perturbation arrows in **(B–D)** can expand (green arrows) or shrink (red arrows) the state space rotation depending on their timing with respect to these state space rotations. In the case of the pendulum, this reduces its maximum angular position and velocity; in the case of neural dynamics, this would reduce the range of neuronal firing rates and theoretically reduce movement amplitude and velocity.

The mechanisms by which BG-Mthal activity influences motor cortical activity are unclear. Implicit in rate/action selection models is the assumption that thalamocortical neurons directly or indirectly activate cortical projection neurons that drive specific muscle activation patterns to generate a complex action (Redgrave et al., 1999; Maia and Frank, 2011; Mink, 2018; Logiaco et al., 2021). An alternative view is that motor cortex functions as a dynamical system whose state depends on its own history and input from other regions (Churchland et al., 2012). The neural “state” is the instantaneous firing rates of cortical neurons (Figure 3). Some of these neurons directly influence downstream effectors (e.g., corticospinal neurons), while others regulate the internal dynamics of the system (e.g., interneurons) (Shenoy et al., 2013). In this view, cortical population activity evolves according to rules determined by intracortical connectivity, and this population activity determines dynamic patterns of muscle activation and movement. Importantly, cortical population activity rotates in the firing rate state space, even for non-periodic movements (Churchland et al., 2012; Vaidya et al., 2015). Larger neural state space rotations are associated with faster movements.

**FIGURE 4:**
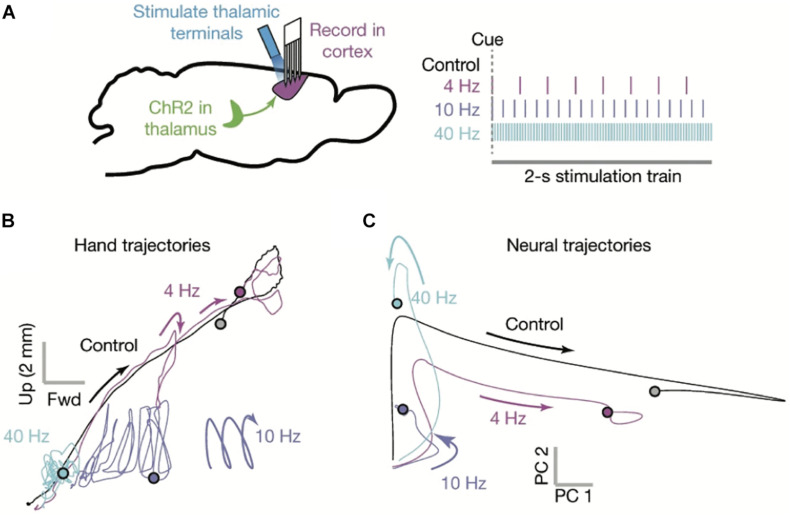
The motor cortex as a dynamical system. **(A)** Multisite single unit recordings were made from primary motor cortex (M1) as mice performed skilled reaching (left). Thalamocortical terminals in M1 (likely from both BG- and CB-Mthal) were optogenetically activated at varying frequencies (right). **(B)** Paw trajectories under different stimulation conditions. Mice made smooth reaches in the absence of stimulation. Paw trajectories were perturbed by 4 Hz stimulation. 10 Hz stimulation created oscillating paw trajectories, reminiscent of action tremor. 40 Hz stimulation severely limited paw movement. **(C)** Neural state space trajectories under different stimulation conditions. 4 Hz stimulation shrunk the state space trajectory, while 10 and 40 Hz stimulation severely truncated the state space rotation. PC1 and PC2 indicate the first two principal components of the neuronal firing rate state space. Adapted with permission from Sauerbrei et al. (2020).

Movement-linked rotational thalamocortical dynamics could explain how the BG regulate movement speed. In a system governed by rotational dynamics, the timing of perturbations with respect to those dynamics is critical. By way of analogy, when a pendulum is at rest, subsequent motion depends on the strength and direction of an initial push, regardless of timing. The resting pendulum may be analogous to motor cortex in a movement-preparatory state (Kaufman et al., 2014, 2016). An abrupt increase in Mthal activity could provide the “push,” with its amplitude determining the size of the subsequent state space rotation and movement speed/amplitude (Gaidica et al., 2018; Catanese and Jaeger, 2021). In this sense, the BG may gate action initiation from a cortical state that has prepared a specific action. Once the pendulum is in motion, its dynamics rotate in the state space described by its angular position and velocity, and perturbation timing becomes critical (Figures 3C,D). Pushing it in the same direction as its instantaneous velocity will accelerate it and increase the size of the state space rotation, but the exact same push 180° later in its swing cycle will slow it and shrink the state space rotation (Figures 3, 4; Dudman and Krakauer, 2016). Instead of interactions between gravity, the cable, and the pendulum bob, cortical system dynamics are generated by interactions among cortical neurons and extracortical input. In particular, BG-Mthal thalamocortical neurons tend to synapse in cortical layer I on the apical dendrites of layer V pyramidal tract (PT) neurons (Herkenham, 1980; Kuramoto et al., 2009, 2015; Guo et al., 2018; Tanaka et al., 2018).

There is experimental evidence that the timing of thalamic input is critical to maintaining cortical dynamics during movement (Sauerbrei et al., 2020). During mouse skilled reaching, optogenetically silencing Mthal disrupts cortical neuronal state space and reach trajectories (Figure 4). Furthermore, optogenetic stimulation of motor thalamocortical terminals has frequency-dependent effects on cortical dynamics and paw trajectories. 4 Hz stimulation disrupts neural dynamics and reach trajectories with each pulse, but there is enough time between pulses for trajectories to partially recover. At 10 Hz, reaching is more impaired, often with an oscillatory component to the paw trajectory. At 40 Hz, reaches are severely impaired. These data indicate that not only is thalamocortical signaling necessary to maintain movement-related cortical dynamics, its timing is critical.

**FIGURE 5:**
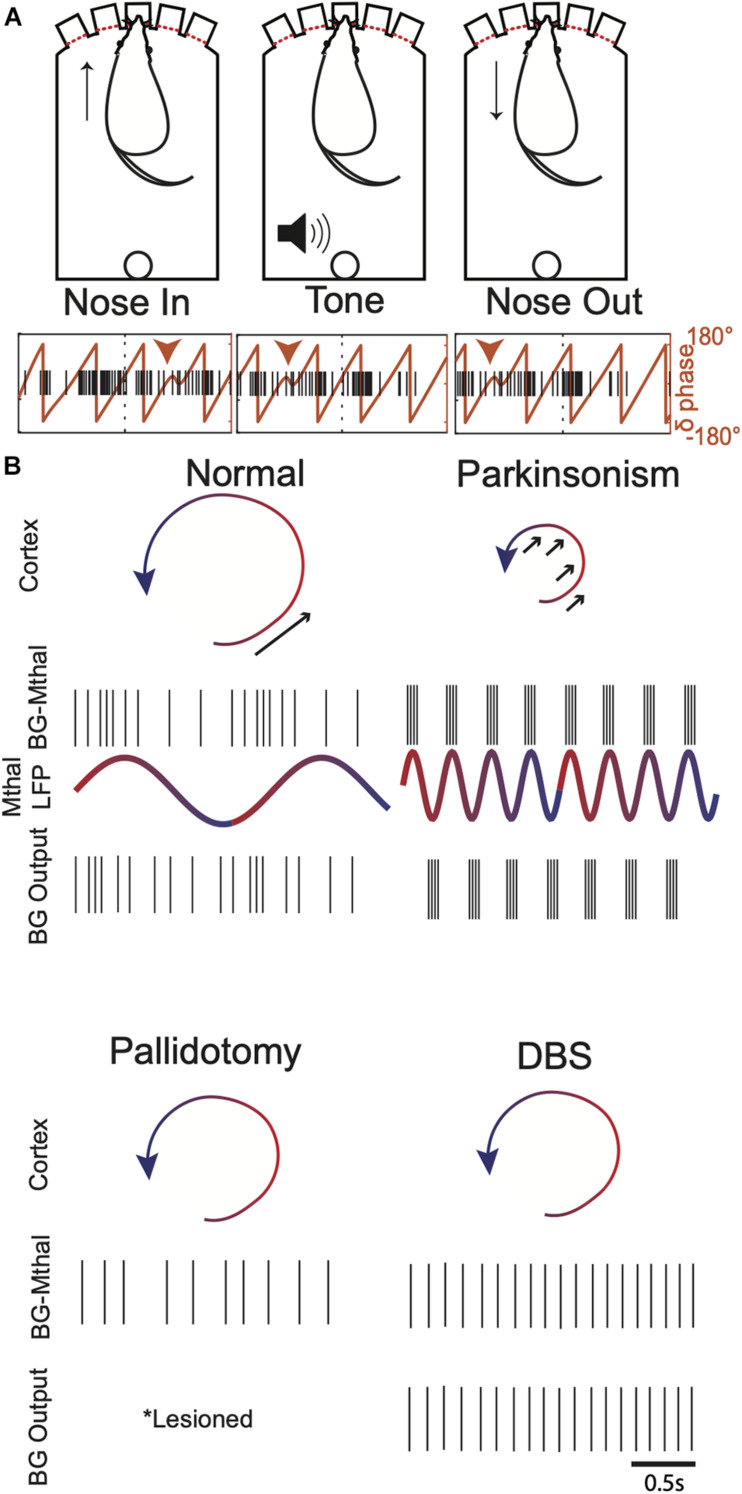
Potential links between Mthal LFP oscillations, cortical dynamics, and single unit BG-Mthal activity. **(A)** Mthal single units are phase-locked to thalamic delta oscillations. Top—behavioral events during a forced-choice reaction time task, in which a rat pokes its nose in a lit port to initiate a trial. A pure Tone then signals the rat to withdraw its nose and poke an adjacent port. Bottom—single trial delta LFP phase and single unit firing during task performance. Note that this unit has a preferred delta phase during which its activity increases. This phase-locking was characteristic of units whose activity predicted the speed of the subsequent nose withdrawal and poke into an adjacent port. Adapted with permission from Gaidica et al. (2020). **(B)** Illustrations of potential pallidal-thalamocortical interactions in healthy subjects, parkinsonism, post-pallidotomy, and during DBS. In healthy subjects, Mthal activity phase-locked to LFP delta oscillations provide well-timed input to enhance cortical state space rotations (arrow). In parkinsonism, thalamocortical neurons synchronized to beta rhythms deliver ill-timed perturbations to cortical dynamics, sometimes enhancing but more often opposing state space rotations (arrows). After pallidotomy, BG-Mthal activity is regulated entirely by cortical input (either directly or via the thalamic reticular nucleus). The BG-Mthal firing pattern is speculative since BG-Mthal activity has not been measured post-pallidotomy, but illustrates the idea that both beta synchrony and appropriately timed pallidal input are lost. During GPi (and to some extent STN) DBS, BG-Mthal units become variably entrained to DBS pulses. This also disrupts beta synchrony but prevents normal pallidal regulation of BG-Mthal spike timing. These single unit patterns are intended to represent typical firing patterns, recognizing that there is variability across the population, even in the synchronized parkinsonian state. Mthal LFPs are omitted from the pallidotomy and DBS panels because these effects are unknown and the model does not make predictions regarding how they should change.

This raises the question of how Mthal “knows” when to push. Synchrony with local field potential (LFP) oscillations may coordinate BG-Mthal activity with cortical dynamics. The phase of cortical and thalamic delta oscillations predicts reaction time (Saleh et al., 2010; Hamel-Thibault et al., 2018; Gaidica et al., 2020; Figure 5) and coordinated corticostriatal low frequency (∼3–6 Hz) LFP oscillations emerge as rats learn single pellet reaching (Lemke et al., 2019). This suggests that low frequency LFPs become coordinated across cortical-BG-thalamocortical circuits as actions are learned, and that the LFP phase at which BG-Mthal spikes occur determines their effect on movement. We speculate that cortical rotational dynamics, which have a frequency of 2–3 Hz in NHPs (Churchland et al., 2012; Kaufman et al., 2016), are reflected in low frequency LFP oscillations synchronized across BG-thalamocortical circuits, at least during movement. Mthal neurons whose activity predicts reaction time and movement speed phase-lock to thalamic delta (∼1–4 Hz), suggesting a mechanism for coordinating Mthal activity with cortical dynamics (Figure 5; Gaidica et al., 2020).

The impact of beta oscillations on movement speed has been studied extensively due to their association with parkinsonism. In addition to predicting reaction times, the phase of cortical and thalamic delta oscillations predicts the amplitude of beta oscillations (i.e., they are phase-amplitude coupled) (Saleh et al., 2010; Lopez-Azcarate et al., 2013; Arnal et al., 2015; Hamel-Thibault et al., 2018; Grabot et al., 2019; Gaidica et al., 2020). Mthal single units whose activity is correlated with movement speed (and also phase-locked to delta oscillations) predict the occurrence of LFP beta oscillations (Gaidica et al., 2020). Thus, in healthy subjects, brief, self-limited bursts of beta oscillations are modulated by the phase of ongoing delta oscillations and the timing of Mthal single unit activity. We suggest that these nested LFP oscillations reflect coordination between Mthal single unit activity and cortical dynamics, which are disrupted in Parkinson Disease.

## BG-Thalamic Interactions in Health and Parkinsonism

This raises the question of how the BG regulate thalamic spike timing. In healthy animals, GPi/SNr neurons fire at relatively high tonic rates (∼70–80 Hz), reflected in flat autocorrelograms (Raz et al., 2000; Bar-Gad et al., 2003). After dopamine depletion, firing patterns become “bursty” with brief periods of high frequency firing (Nini et al., 1995; Boraud et al., 1998; Raz et al., 2000; Soares et al., 2004). As in STN and cortex (reviewed above), GPi/SNr neurons tend to fire in oscillatory patterns and entrain to LFP oscillations (Chan et al., 2011; Brazhnik et al., 2012). In addition to LFP entrainment, GPi neurons become synchronized with each other. Cross-correlograms between GPi neurons are almost universally flat in healthy NHPs (Bar-Gad et al., 2003), but develop significant correlations after dopamine loss (Nini et al., 1995; Heimer et al., 2002, 2006). As in other brain regions, these changes normalize at least partially with treatments that improve symptoms including levodopa (Heimer et al., 2002, 2006) and DBS (Hashimoto et al., 2003; Hahn et al., 2008; Malekmohammadi et al., 2018). While these changes are consistently observed in advanced parkinsonism, studies in progressive models of nigral degeneration generated conflicting results regarding which physiologic changes correlate best with parkinsonism across disease stages (Leblois et al., 2007; Devergnas et al., 2014; Connolly et al., 2015a,b; Muralidharan et al., 2016; Escobar Sanabria et al., 2017; Willard et al., 2019).

Several potential mechanisms of BG-thalamic interactions have been proposed (Goldberg et al., 2013). The first is a rate model view, in which tonic GPi activity suppresses BG-Mthal activity until it is released to initiate action. Arguments against this idea have been detailed above (see *Evidence Contradicting Rate Models*), though rate models may apply under specific conditions (for example, transitioning from rest to movement or during especially strong direct/indirect pathway activation) (Kravitz et al., 2010; Schmidt et al., 2013; Gaidica et al., 2018). The second is a rebound model that focuses on low threshold spike (LTS) bursts generated by T-type Ca^2+^ channels after hyperpolarization. Transiently elevated inhibition from the BG could provide the hyperpolarization necessary for LTS bursting, though LTS bursts appear to be uncommon in healthy animals during wakefulness (Bosch-Bouju et al., 2014). The third mechanism is entrainment of BG-Mthal firing to pallidal inputs. In the songbird, pallidal (Area X) spikes inhibit BG-Mthal (DLM) units for several milliseconds, after which their excitability increases. During epochs of strong cortical input (e.g., singing), BG-Mthal spikes become tightly aligned to pallidal spikes without significantly affecting mean firing rates (Figure 2; Goldberg and Fee, 2012).

BG-thalamic interactions may operate mostly in “entrainment” mode in healthy animals, with the “rebound” mode becoming more important after dopamine depletion. LTS bursts may become more frequent due to burst-firing at the GABAergic BG output, as well as changes in the intrinsic properties of BG-Mthal neurons (Edgerton and Jaeger, 2014; Bichler et al., 2021). Artificial excitation of BG afferents in mouse BG-Mthal can generate LTS bursts in slice and *in vivo*, which depends on the presence of T-type Ca^2+^ channels (Edgerton and Jaeger, 2014; Kim et al., 2017). These bursts are enhanced and linked to motor dysfunction in SPR-knockout mice, which are dopamine-deficient due to defects in tetrahydrobiopterin synthesis (Kim et al., 2017). Furthermore, models suggest that synchronous BG output is especially effective at generating rebound bursting (Nejad et al., 2021).

Transitions between BG-Mthal firing modes in different dopaminergic states are plausible, but there are relatively few studies of thalamic physiology in the dopamine-depleted state. Results have been inconsistent regarding the importance of thalamic burst-firing and beta synchrony in parkinsonism. Increased BG-Mthal burst firing has been observed in MPTP-treated compared to healthy NHPs (Pessiglione et al., 2005), but this was not true in all studies or in 6-OHDA treated rats (Bosch-Bouju et al., 2014; Kammermeier et al., 2016). BG-Mthal burst-firing was also observed in humans with PD (especially during tremor), but cannot be directly compared to healthy controls (Zirh et al., 1998; Magnin et al., 2000; Molnar et al., 2005). In NHPs, findings regarding oscillatory activity are also inconsistent. Strong low frequency LFP oscillations (3–9 Hz) coherent with frontal EEG signals were observed in humans with PD, with a smaller peak in beta power coherent with EEG leads over ipsilateral motor cortex (Sarnthein and Jeanmonod, 2007). In contrast, higher frequency (∼15 Hz) LFP oscillations were observed in BG-Mthal of MPTP-treated NHPs. Single BG-Mthal units became entrained to these “low beta” oscillations, and themselves exhibited low frequency oscillatory firing patterns (Kammermeier et al., 2016). In hemiparkinsonian rats, LFP power increased at higher frequencies (∼30–40 Hz), also with BG-Mthal single-unit entrainment to those rhythms (Brazhnik et al., 2016). More impressively, correlations between the activity of BG-Mthal neurons markedly increased after dopamine-depletion, which was associated with loss of specificity for joint movements (i.e., neurons responded to movement across multiple joints instead of just one) (Pessiglione et al., 2005).

While the key links between thalamic physiology and parkinsonism are not fully defined, BG-Mthal units consistently develop low frequency (< ∼20 Hz) synchronous oscillatory firing after striatal dopamine loss in NHPs. Periodic synchronized BG-Mthal activity could disrupt cortical rotational dynamics, similar to artificial stimulation of thalamocortical terminals (Sauerbrei et al., 2020; Figures 3–5). While a single well-timed perturbation during a rotational cycle could appropriately enlarge or shrink cortical state space rotations, periodic high frequency perturbations would repeatedly disrupt evolving dynamics. In particular, there seems to be something special about synchronized neuronal activity at beta frequencies, suggesting that they are well-timed to shrink cortical state space rotations associated with movement.

## Resolving the “Paradoxes of Stereotaxic Surgery”

Considering motor cortico-thalamocortical circuitry as an independent dynamical system modulated by BG output could resolve the apparent paradoxes associated with lesions and DBS (Figure 5). Loss of BG output (i.e., pallidotomy) would allow thalamocortical dynamics to evolve unperturbed. The cost of removing BG output would be loss of appropriate BG-mediated modulation of thalamocortical circuits. In the pendulum analogy, the ability to increase the pendulum’s momentum would be lost, but at least it would not be slowed by poorly timed perturbations. This could explain why slow patients move faster, and fast patients move slower after pallidotomy (Bastian et al., 2003).

DBS of the STN and GPi have complex effects on BG-Mthal and cortical activity patterns. In MPTP-treated NHPs, STN DBS did not reduce BG-Mthal bursting, but did reduce single-unit oscillatory power between ∼3–30 Hz (Xu et al., 2008). Three studies examined the effects of GPi stimulation on BG-Mthal activity in NHPs, finding variable changes in BG-Mthal firing rates at the single unit level, no change in burst frequency (though some changes in burst characteristics), and reduced single-unit entrainment to beta oscillations (Agnesi et al., 2015; Kammermeier et al., 2016; Muralidharan et al., 2016). The latter could be responsible for reductions in motor cortical beta oscillations (single-unit and LFP) during GPi DBS (McCairn and Turner, 2015; Wang et al., 2018). Two studies found BG-Mthal entrainment to GPi stimulation, which inhibited a large fraction of BG-Mthal units for several milliseconds with each stimulus pulse (Agnesi et al., 2015; Muralidharan et al., 2016). However, responses were variable at the level of individual neurons, including decreased, increased, or multimodal firing patterns in response to each stimulus pulse.

While these data do not reveal a clear singular mechanism by which high frequency DBS improves parkinsonism, they suggest that an important therapeutic effect is to disrupt beta synchrony among BG-Mthal neurons. High frequency STN or GPi stimulation creates narrow time windows during which thalamocortical neurons can be excited by corticothalamic input, preventing BG-Mthal neurons from becoming entrained to synchronous beta frequency input from GPi. Indeed, GPi DBS creates a peak in BG-Mthal single-unit power spectra near the stimulation frequency (Muralidharan et al., 2017). As in the case of pallidotomy, the cost of preventing transmission of beta synchrony into BG-Mthal would be loss of BG control over thalamocortical spike timing.

## Summary and Future Directions

We suggest that delta frequency LFP oscillations reflect movement-related cortical rotational dynamics. Single-unit BG-Mthal activity phase-locked to delta oscillations could speed or slow movement, depending on the timing with respect to cortical dynamics. A major function of the BG may be to adjust the strength and timing of BG-Mthal spiking with respect to cortical dynamics depending on task demands.

Like rate models, this hypothesis makes testable predictions. First, cortical state space rotations should align consistently with delta LFP phase. Second, phase entrainment of BG-Mthal spikes to LFP delta oscillations should vary with movement speed. Third, artificially manipulating BG-Mthal spike timing with respect to delta oscillations should alter movement speed. Finally, the same movement should generate qualitatively similar cortical dynamics with different sized state space rotations under parkinsonian and treated conditions (e.g., with levodopa or DBS).

If this model is correct, it still leaves several open questions. First, it is not clear how striatum, globus pallidus, and the subthalamic nucleus interact to optimize GPi spike timing. Second, the neural mechanisms by which BG-Mthal (or CB-Mthal) activity influence cortical dynamics are unknown. BG-Mthal thalamocortical neurons project to both premotor and primary motor regions, where they tend to synapse in cortical layer I on the apical dendrites of layer V pyramidal tract (PT) neurons (Herkenham, 1980; Kuramoto et al., 2009, 2015; Guo et al., 2018; Tanaka et al., 2018). Corticothalamic projections to BG-Mthal arise at least in part from layer V pyramidal neurons in premotor cortex, forming a closed loop with premotor-projecting thalamocortical neurons that appears to be segregated from primary motor cortical-thalamic loops (Guo et al., 2018). While layer II/III-specific CB-Mthal projections to primary motor cortex have been defined (Hooks et al., 2013; Yamawaki and Shepherd, 2015) the circuits linking BG-Mthal and primary motor cortex are less well-characterized. Furthermore, premotor and motor cortical layer VI neurons project to the inhibitory thalamic reticular nucleus, which in turn projects to motor thalamic regions. How this complex circuitry allows the BG to influence motor cortical dynamics and, ultimately, motor output is unknown.

It is also not clear why LFP frequencies that are enhanced by dopamine loss are different across species, making it difficult to determine which physiologic changes are most relevant to humans. While basic features of BG-thalamocortical communication are preserved from rodents to primates, there are important differences. Primate Mthal has inhibitory interneurons that are absent (or at least very sparse) in rodents (Jones, 1981). Dopaminergic innervation of Mthal is also much stronger in primates than rodents (Sanchez-Gonzalez et al., 2005). Perhaps more importantly, rodent BG output is primarily from SNr with a much smaller contribution from the entopeduncular nucleus (the rodent analog of GPi). The degree to which these differences are meaningful for interpreting rodent results in the context of human disease is uncertain.

This model also does not readily explain hyperkinetic disorders like dystonia or chorea. One possibility is that aberrant thalamocortical communication may not simply shrink or expand cortical state space rotations, but force them onto entirely new trajectories. For example, pushing a pendulum out of its plane of motion would lead to completely new dynamics. Unfortunately, there are few animal models in which to study the thalamic physiology of hyperkinetic disorders other than levodopa-induced dyskinesias. One important clue may come from differences between STN and GPi DBS. Despite mostly similar clinical effects, STN DBS causes dyskinesias which usually wane over time, while GPi DBS suppresses them. Whether this is due to distinct effects on BG-Mthal activity or extrathalamic effects is unknown.

The BG also influence motor function through non-thalamic pathways for which we have not accounted. BG output flows to brainstem nuclei including the superior colliculus, which is important for orienting movements (e.g., attending to a loud noise or bright light) (Park et al., 2020). The STN projects to pontine nuclei that project to the cerebellum (Bostan et al., 2010) providing a potential mechanism for STN DBS to affect tremor. STN DBS can also activate motor cortical neurons by retrograde activation of hyperdirect pathway axons (Gradinaru et al., 2009; Walker et al., 2012; Miocinovic et al., 2018), though recent studies suggest that this may not be a critical mechanism (Johnson et al., 2020; Yu et al., 2020). The relevance of each of these projections to the pathophysiology of parkinsonism (and other movement disorders) and the effects of DBS remain to be determined.

We have suggested a novel framework in which to understand BG-thalamocortical interactions that is almost certainly oversimplified. The concept of BG modulation of thalamic spike timing has been suggested previously (Goldberg et al., 2013; Dudman and Krakauer, 2016). Here, we add the idea that a major role of BG-Mthal is to regulate the size of cortical state space rotations, and therefore movement speed/amplitude, with well-timed spikes. The timing of BG-Mthal input to motor cortex with respect to cortical dynamics (reflected in LFP delta oscillations) determines whether movements will be sped or slowed, and the strength of BG-Mthal signaling determines the magnitude of the effect. This model is consistent with known pathophysiologic changes in parkinsonism, and may explain the apparently paradoxical clinical effects of BG lesions and DBS. Importantly, this model makes specific testable predictions, much like the rate model which has facilitated impressive progress in understanding BG influences on motor control over the last 30 years.

## Author Contributions

JM and DL contributed to the format and writing of the review and to the selection and design of the figures. Both authors contributed to the article and approved the submitted version.

## Conflict of Interest

The authors declare that the research was conducted in the absence of any commercial or financial relationships that could be construed as a potential conflict of interest.

## Publisher’s Note

All claims expressed in this article are solely those of the authors and do not necessarily represent those of their affiliated organizations, or those of the publisher, the editors and the reviewers. Any product that may be evaluated in this article, or claim that may be made by its manufacturer, is not guaranteed or endorsed by the publisher.
